# Care coordination, consistency and continuity: the case of the key worker role in children’s cancer care

**DOI:** 10.1080/17482631.2022.2092958

**Published:** 2022-06-26

**Authors:** Ana Martins, Susie Aldiss, Rachel M Taylor, Faith Gibson

**Affiliations:** a UCLH Cancer Clinical Trials Unit, University College London Hospitals, London, UK; b School of Health Sciences, Faculty of Health & Medical Sciences, University of Surrey, Guildford, UK; c Centre for Nurse, Midwife and Allied Health Profession Led Research (CNMAR), University College London Hospitals NHS Foundation Trust, London, UK; d Centre for Outcomes and Experience Research in Children’s Health, Illness and Disability (ORCHID), Great Ormond Street Hospital for Children NHS Foundation Trust, London, UK

**Keywords:** Key worker, nurse specialist, care provision, children, cancer, evaluation, nursing

## Abstract

**Purpose:**

The overall aim was to evaluate the key worker role across principal treatment centres for children with cancer in England, Wales and Scotland.

**Methods:**

Mixed-methods case study gathering data from multiple perspectives using questionnaires, interviews, focus groups and reports/performance documents over a two-year period. Framework approach was adopted to analyse transcripts and documentary data.

**Results:**

Participants included: 22 nurse specialist key workers, 103 parents, 85 professionals and 10 children/young people. Qualitative and quantitative data were woven together, to best illuminate key worker services. Four main models of care were described as well as the context of care and process of care. Key working effectiveness centred around three pillars: care coordination; expert knowledge, experience and expertise; relationship. These were essential to improved family experience, emotional wellbeing, and delivery of individualized care closer to home.

**Conclusions:**

The role is complex and diverse, responding to local needs. Certain conditions, (e.g., high caseload) placed limits on enacting the three pillars, diminishing the positive experience of families. When they worked well, key workers reduced the fragmented nature of services and families placed great value on keeping the same key worker from diagnosis into long-term care. Retaining these roles, where already in place, or including, if not, we would recommend, factoring into budgets to sustain and expand such roles.

## Introduction

A known challenge for healthcare systems around the world is how to deliver high-value, effective care, while managing the increasing financial costs of care (Jessup et al., 2020). The usefulness of any given model of service delivery is based on its ratio of benefits and harms relative to the actual costs, often informed by economic evaluation. Internationally, in children’s cancer services, although there are many different approaches to service delivery, more often informed by minimal evaluation, they are all underpinned by a shared ethos of interdisciplinary care. Therefore, learning from one service to another is possible, particularly where that new learning is informed by stakeholders, that includes patients, family members and healthcare professionals. In 2009, with a steer from Young Lives vs Cancer (formally CLIC Sargent), a children’s cancer charity in the UK, a new model of care was recommended. Core to this model was the following: that every child and family should have a key worker responsible for coordination of care and support in the community; their needs would be systematically assessed and reassessed using the Common Assessment Framework (Snowden et al., 2015); they would easily be able to access support/advice at any time; they would be given information to enable them to understand/manage their illness, empowering them to make informed choices about their care; they would receive a tailored package of care delivered by health, education and social care professionals (CLIC Sargent, 2009). In response to these recommendations, across the UK, clinical posts were established as part of the Young Lives vs Cancer key worker project. Reported here is an evaluation of these posts.

## Background

Service delivery in England, Scotland and Wales for children with cancer is based in 19 specialized principal treatment centres (https://www.cclg.org.uk/In-hospital/Specialist-hospitals). Many of these centres have developed patterns of work specific to their geography. A model delivering care more local to a child’s home, known as “shared care”, is offered in some centres. This array of models of care delivery, in a small number of highly specialized centres, has required the involvement of a range of professionals across primary care (General Practitioners, health visitors), secondary care (local district general hospitals) and tertiary care (principal treatment centres). This wide-ranging scope of services can make it difficult for families to find their way through the complex healthcare continuum and obtain the support they need. This can result in a lack of integration and continuity of care (Freeman & Rodriguez, 2011). One solution to this is to maximize care coordination, to improve processes designed to streamline and navigate the system, creating a seamless flow across the care continuum (Dixit et al., 2021). Patient navigation, is a process whereby families receive support from an individual, who could guide them throughout the cancer care continuum (Cook et al., 2013). In adult cancer care, navigation has a strong evidence base for improving follow-up care, adherence to treatment, and care documentation (Dickerson et al., 2020). Navigation and care coordination are central to the role of the key worker, a role first developed in adult cancer care in the UK (Ling et al., 2017). Often this role is fulfilled by skilled nurses, usually a clinical nurse specialist, acting as a type of “broker”, to provide instrumental and relational functions and processes to support patients, and families, quickly identifying emerging issues and improving the overall patient experience (National Cancer Action Team, 2012). The positive contributions to adult cancer care made by clinical nurse specialists and their role as key workers, across the range of diseases, is well documented, revealing their responsibilities to be multifaceted and diverse (Kerr et al., 2021). The key worker role has been implemented in children’s cancer care, but how it is perceived by other staff members, patients and families, has so far not been examined. The Young Lives vs Cancer specialist nurse key worker role project provided this opportunity.

Our evaluation study was designed to address this gap in our knowledge, to consider the varied approach to implementation of the key worker role, that could impact upon sustainability. The Young Lives vs Cancer key worker role was established to provide holistic and individualized care to the child and family, facilitating safe care close to home. In the first part of our study we described these roles as diverse, clearly responding to local need, and firmly embedded within care settings (Martins et al., 2016). We sought to expand this description, focussing on the different patterns of provision across services, and the determinants for success that would indicate core requirements to achieve well-coordinated transitions, for example, from hospital to home, between tertiary, secondary and primary care. We have previously reported on the role in terms of when it works well and when it is more challenging (CLIC Sargent, 2015), here we report on the detail of the model of key working in children’s cancer services.

## Aim

To evaluate the key worker role and its impact on: patient and family experience; parental emotional wellbeing; the delivery of care closer to home and its benefits for children and their families.

## Methods

### Design

We carried out an evaluation using case studies: specifically, an “intrinsic” case study, where the case itself was the key worker role. Other participants (parents, children, young people, and professionals) were linked to each case and their practice. Case study research takes a holistic approach (it considers the case within its context) and is characterized by a convergence of diverse sources of data, which provide a means of considering the multiple elements likely to shape and influence the case, in this context the key worker role (Stake, 1995). To understand and reveal complexities of the case, we collected multiple sources of data that centre on the key workers’ role. Reporting of findings followed the Good Reporting of a Mixed-methods Study (GRAMMS) guidelines (O’Cathain et al., 2008). The GRAMMS checklist is available as a supplementary file.

### Setting and participants

**Figure 1.**
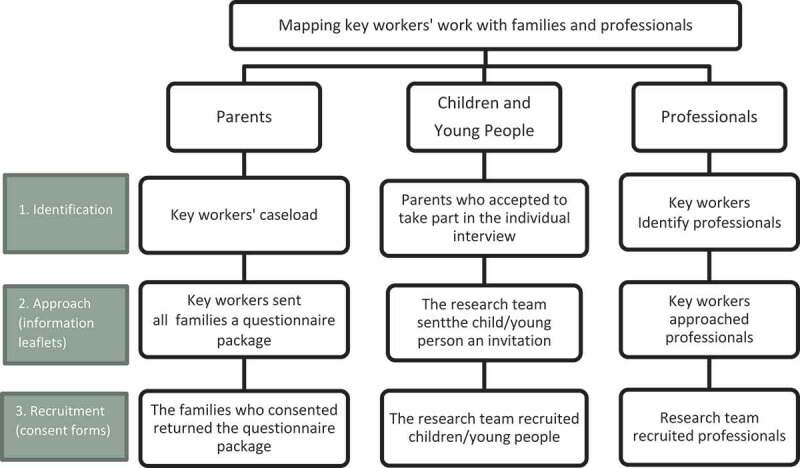
Overview of the recruitment strategy.

Children’s cancer care in the UK is delivered at 19 tertiary centres, referred to as principal treatment centres. Our study was conducted in these centres across England, Wales and Scotland, where 24 specialist nurses were funded in the key worker role; three did not have a caseload, they were funded to work solely in education; three of the centres supported more than one role. Our study involved the key workers, recruiting also from their caseload of parents, children and young people under the age 16 years. Key workers identified professionals, individuals who worked closely with the key worker within different settings, including hospital and community (such as children’s community nurses, medical doctors, allied health professionals and social workers). Our approach to recruitment is outlined in Figure 1.

### Data collection

**Figure 2.**
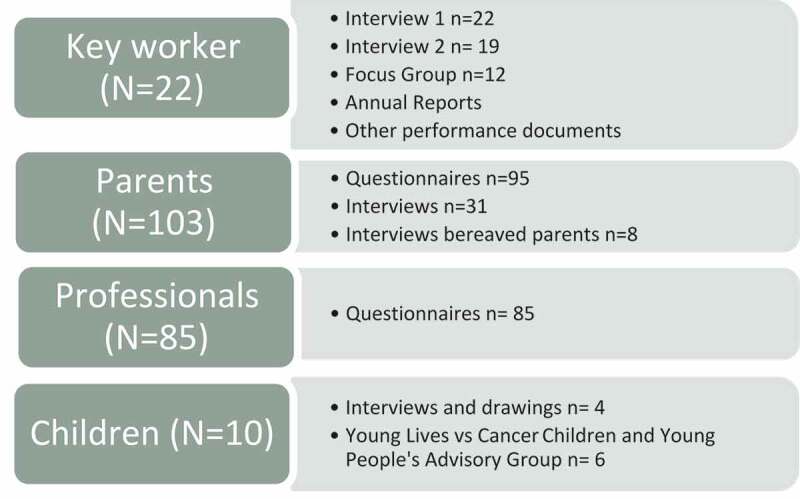
Participants and data sources.

The key worker service is a complex healthcare programme of care. Thus, a range of data were required to best illuminate this role and its points of impact. We used a combination of semi-structured individual interviews, focus group and questionnaires, collecting evaluation/performance data over a two-year period, 2013 to 2015. The aims, data collection methods and outcomes explored by each method are shown in Table I. Figure 2 presents participants alongside data sources.

**Table I.** Aims and data collection methods

Objectives	Children/ Young People		Parents		Key workers		Professionals			Young Lives vs Cancers’ anonymised data	
Evidence whether there is an improvement in patient and family experience as a result of nursing key worker support.	Interview	Participatory age appropriate methods were used in these interviews. Questions related to aspects of the context, the intervention (what was expected the key worker responsibilities and competencies) and the outcomes.	More Than My Illness Questionnaire Package for Parents Interview	Interviews aimed to gain an in-depth understanding of families’ experience of the key worker services. Questions related to aspects of the context, the intervention (what was expected the key worker responsibilities and competencies) and the outcomes. Parents’ questionnaire package^1^ - validated questionnaires as well as investigator lead questionnaires covering these outcomes: Parental needs (Beresford, 1995; Quine & Pahl, 1989; Sloper & Turner, 1992) Impact of key worker on quality of life (Mukherjee, Beresford, & Sloper, 1999) Stress and overall satisfaction with the key worker service - as well as the responsibilities associated to the role - aspects of key working (Mukherjee et al, 1999) Contacts with the service; professionals signposted by the key worker Parents empowerment Key worker communication and coordination Measure of process of care (King, Rosenbaum, & King, 1995) Key worker specific tasks	Interview	An in-depth exploration of key workers’ role and the context of their practice were explored in the interviews and focus group with key workers. Questions related to aspects of the context, the intervention (what was expected, key worker responsibilities and competencies) and the outcomes. The second interview focused on exploring topics that needed further development following the first interview and on how the role had developed since the first interview.		Questionnaire	The questionnaire explored: Perceptions of key worker services Frequency and nature of contact The ways in which the key worker role has affected families’ relationships with the professionalr’s service Views on advantages and limitations of key working Knowledge about the key worker service Suggestions for service development If best practice and learning has been adopted by other practitioners within local terms.	performance data	Patient focused meetings Contact with professionals within (e.g. social worker) and outside (e.g. GP) the hospital Number of home visits attended and arranged How many times the care was delivered in specific settings (e.g. school) Discharge planning
Evidence whether children/young people and their families have improved emotional well-being as a result of nursing key worker support.	Interview		More Than My Illness Questionnaire Package for Parents Interview		Interview			Questionnaire			
Evidence whether children/young people are better able to participate in education, employment and training as a result of nursing key worker support.	Interview		More Than My Illness Questionnaire Package for Parents Interview		Interview			Questionnaire		performance data	
Evidence whether children and their families are better able to spend more time safely at home during treatment as a result of nursing key worker support.	Interview		More Than My Illness Questionnaire Package for Parents Interview		Interview			Questionnaire		performance data	
Identify obstacles to providing care closer to home that may or may not be overcome by the key worker role.			Interview		Interviews Focus group			Questionnaire			
Evidence whether best practice and learning have been adopted by other practitioners within local terms.					Interview			Questionnaire			

#### Measures

##### Parents

The “more than my Illness” package of questionnaire explored 11 separate domains (Table II). This suite of questionnaires took approximately 45 minutes to complete.

**Table II.** *More than my illness* questionnaire package for parents.

Construct and questionnaire	Details
Parental needs [Alpha reliability was 0.85 (N = 129)]	A 21-item scale of parental needs. Comprises items needs common to parents, such ‘spending more time with my partner’, ‘having more time with my other children’ and ‘help getting my child to sleep better’. Items are rated on a three-point scale, of ‘Getting enough help’, ‘Need help’ or ‘Help not needed’.The scale has been used in previous research (Beresford, 1995; Quine & Pahl, 1989; Sloper & Turner, 1992).
Aspects of key working [Alpha reliability was 0.95 (N = 163)]	A 12-item scale was used measuring how much the family’s key worker performed various aspects of the key worker role. Items in the scale are based on earlier research on the role of the key worker (Mukherjee et al., 1999). Examples of items are: emotional support, information about your child’s condition, information about services, advice, identifying the needs of all family members and addressing the needs of all family members. Two items were added to the original scale—‘signposting you to other services’ and ‘other’. Respondents rated the items as ‘Not at all’, ‘Some’ or ‘Very much’, according to how much support they received from their key workers on each of these.
Impact of key worker on quality of life [Alpha reliability was 0.85 (N = 173)]	A seven-item scale will be used to measure the effects of having a key worker on parental Quality of Life (QOL). This scale had been used in previous research (Mukherjee et al., 1999) and incorporated items such as ‘My physical health or well-being (for example, sleep, rest, exercise)’, ‘My emotional/mental health (for example, stress, anxiety, depression)’ ‘Time to myself (for example, work, studies, interests)’, ‘My relationships’, and ‘My financial or material circumstances (for example, income, housing)’. Participants identified if the key worker had an impact on these areas of their lives over the past six months.
Stress	One item measure of how the contacts with the key worker service affected the amount of stress experienced by parents from “considerably reduced my stress” to “considerably increased my stress”.
Contacts with the service	Parents were asked to identify the frequency of contact and if they would like more, the same or less contact and who initiated the contact (the key worker or the parent).
Professionals signposted by the key worker	A list of professionals is presented with three options for each professional listed: I/my child have seen; signposted by the key worker and both.
Parents empowerment	Three questions to measure the key worker role in empowering the parent. Participants rated each question from 1 (never) to 4 (always).
Key Worker Coordination and communication with parents	Seven questions measuring how often the parent was confused or unsure who to contact and the roles as well as waiting for appointments and information sharing between professionals. Respondents rated the items from 1 (Never) to 5 (Always).
Measure of Processes of Care [Alpha reliability was 0.99 (N = 31)]	The anglicized version (McConachie & Logan, 2003) of the Measure for Processes of Care (MPOC, (King et al., 1995) was used. The MPOC is defined as a means to assess family-centred behaviours of professionals in services for disabled children and is a self-report measure of parents’ perceptions of the extent to which specific behaviours of care professionals occur. Respondents were asked to rate each item on a four-point scale from one Never to four Always, or as ‘not applicable’. For the purposes of this study, four questions specific to children with disability were removed (total 51-item scale).
Key worker role specific tasks	Fifteen tasks included in the description of the key worker role were listed and participants were asked if in the last 6 months the key worker had performed each of the tasks using one of the three options to answer “yes”, “no” and “n/a”
Satisfaction with the key worker service	One item measured how satisfied the respondent was with the key worker service. The question was “Overall, how satisfied are you with the key worker service you receive?” The question was rated on a four-point scale from “Very satisfied” to “Not at all satisfied”

##### Professionals

Completed an investigator designed questionnaire. A 5-point scale, 1 (never) to 5 (always) across the following areas: perceptions of key worker services; frequency and nature of contact; how the role had affected families’ relationships with the professional’s service; opinions on advantages and limitations of key working, for children and families and the professional’s service; knowledge about the key worker service; suggestions for service improvement; what best practice/learning had been adopted by other practitioners within local teams.

### Ethical considerations

Approvals were granted by an NHS Research Ethics Committee (Reference: 12/WM/0365). Data were collected in 19 principal treatment centres for whom approval to approach staff, families and patients had been granted. Written consent to participate was sought from parents. Children signed an assent form and a parent signed a parental consent form. Written consent was not sought from professionals or key workers as this was not a requirement in the UK in 2015. Clarity regarding reporting of our study was essential at the outset, to assure all potential participants that only anonymized data would be reported, with reference to “generic key worker roles”, across a service; there would be no mention of individual services or specific roles. Key workers had no knowledge of which parents/carers/children participated.

### Data analysis

The core element of our analysis was the qualitative data generated through interviews and documentary data. The interviews and focus group were transcribed verbatim for analysis. Quantitative data were used as complementary to the qualitative data. Descriptive statistics were used to report those aspects of data which were amenable to this approach. Framework approach was used to analyse interview/focus group transcripts and documentary data: an approach known to be both flexible and rigorous during team analysis (Parkinson et al., 2016). Data were charted and sorted into a framework to facilitate comparisons and interpretation of the key ideas and emergent themes. Analysis was undertaken by two researchers (SA, AM) and validated by a third (FG). A preliminary framework was developed by AM, this evolved during analysis of the initial transcripts by SA and AM. Briefly, the analytical steps included: (1) familiarization, (2) identifying a thematic framework, (3) indexing, (4) charting and (5) mapping and interpretation (Ritchie et al., 2013).

### Validity, reliability and rigour

To ensure rigour and consistency, the same researcher, with significant experience in qualitative research, undertook all data collection (AM). A semi-structured interview guide was used and questions were piloted. Two experienced researchers (AM and SA) undertook the analysis and met regularly together and with a third researcher (FG) to discuss ongoing analysis and resolve discrepancies. The use of framework analysis enabled the researchers to review the coding to check for accuracy of the interpretation.

The questionnaire package for parents was based on questionnaires used in similar evaluation research (Greco et al., 2005). Previously validated questionnaires were used where available, see Table II for alpha reliability values. The questionnaire for professionals was investigator designed for this evaluation, this was non-validated and was piloted prior to use. It was informed by Greco et al.’s (2005) evaluation of key worker services for children with disabilities and Carter et al.’s (2010) evaluation of the WellChild Children’s Nurse programme.

## Results

We present a synthesis of the key worker service, across 19 sites. This synthesis is a result of an in-depth description of each individual key worker service gathered from multiple data sources. We present first a summary of the participants in our study (for more details see, CLIC Sargent, 2015), followed by the models of key working, and the context of care. The final section of our results focusses on the process of care, and presents an expanded description of the three pillars: care coordination; expert knowledge, experience and expertise; and relationship, first highlighted in CLIC Sargent (2015). Relevant data sources are drawn upon, weaving together qualitative and quantitative data, to best illuminate key worker services.

### Participants

The key workers were attached to principal treatment centres and were, in the main, specialist nurses experienced in children’s cancer care. Professional backgrounds included: outreach nurse specialist (n = 14); clinical nurse specialist/specialist practitioner (n = 4) and other (n = 3). Eighty-five professionals who worked closely with the key worker and families from all sites took part, these included: 27 nurses (including community nurses and lead cancer nurses); 25 doctors (including shared care consultants and palliative care consultants); eight allied health professionals (including occupational therapists, dietitians, physiotherapists and radiographers); 12 social workers and ten professionals from other roles (including pharmacists, play specialists, psychologists, teachers, service managers). Some professionals worked in the same hospital as the key worker and others worked outside). One hundred and three parents participated and 10 children/young people (see, Table III for details of the 95 parent/carers who completed the questionnaires). Participating bereaved parents included six mothers and two fathers.

**Table III.** Demographic details of parent/carers who completed the questionnaires (n = 95).

Details	Response	n (%)
Participant	Mother	81 (85%)
	Father	10 (11%)
	Other	2 (2%)
	Missing data	2 (2%)
Parent/carer age	20–29 years	3 (3%)
	30–39 years	33 (35%)
	40–49 years	40 (42%)
	50–59 years	9 (9%)
	Missing data	11 (12%)
Parent/carer ethnic background	White	86 (91%)
	Asian	5 (5%)
	Other	1 (1%)
	Missing data	3 (3%)
Number of children	One child	19 (20%)
	Two children	39 (41%)
	Three children	27 (28%)
	Four or more children	7 (7%)
	Missing data	3 (3%)
Marital status	Married	78 (82%)
	Separated or divorced	8 (8%)
	Single	7 (7%)
	Missing data	2 (2%)
Gender of child with cancer	Female	40 (42%)
	Male	53 (56%)
	Missing data	2 (2%)
Age of child with cancer	0–2 years	3 (3%)
	3–5 years	27 (28%)
	6–8 years	22 (23%)
	9–11 years	10 (11%)
	12–14 years	16 (17%)
	15–18 years	15 (16%)
	Missing data	2 (2%)
How long the family have had a keyworker	1–6 months	21 (22%)
	7–12 months	29 (31%)
	1–2 years	25 (26%)
	2–3 years	13 (14%)
	Over 3 years	3 (3%)
	Missing	4 (4%)

Interviews with parents/bereaved parents lasted between 30 minutes and two hours and 16 minutes. Interviews with key workers lasted 45 minutes to one hour and 55 minutes. The focus group with key workers lasted one hour and was attended by 12 key workers. Four children took part in short interviews and six participated as part of an advisory group.

### Models of care

Differences between how the roles were operationalized transpired, during the interviews with key workers and via the evaluation/performance data collected. Roles could be described along a continuum of “in-reach” and “outreach”, with the presence/absence of home visits and direct delivery of clinical care, distinguishing between these roles across four different models of care (Martins et al., 2016).

#### Model 1-outreach

Some of the key workers were previously outreach nurse specialists, as key workers they continued to support families in the community, undertaking home visits:

I would be their key worker for their home situation rather than the key worker in the hospital (Key worker 19, interview)

… … … going off to either do home visits to children to do blood tests, if they’re due chemotherapy, or to give chemotherapy at home (Key worker 17, interview)

#### Model 2-in-reach with home visits

In this model, key workers were based in the hospital and they also carried out home visits occasionally; for example, they might do a home visit after discharge, or they might be involved in school visits:

I would see them when they’re in hospital and then I would, either go out myself and see them at home, or the POONS [Paediatric Oncology Outreach Nurse Specialist] would go out and see them at home” (Key worker 12, interview)

Joint home visits with community teams were valued by professionals, with key workers being very clear on their purpose:

(… … .) what we would try to do is do a home visit with the community nurse, quite early on in the treatment … … … … .to say to the family, so and so will be visiting you once a week to flush your line, so they build up that relationship (Key Worker 6, interview)

#### Model 3-in-reach

Here, key workers were mainly based in the hospital with a greater in-reach focus:

I meet the children at diagnosis when they are on the ward, and then I may see them when they come in to outpatients (…) I don’t see them in their homes (Key Worker 13, interview)

Evaluation/performance data showed the range of ways key workers maintained connections and support of families, even when hospital based, by maintaining contact with other professionals outside of the hospital, such as General Practitioners, shared care nurses, community nurses, and within the hospital, such as physiotherapists, occupational therapists, statutory social worker, Young Lives vs Cancer Social worker, and using email, texts and phone calls to reach other professionals involved in the child’s care. These approaches were valued by professionals, where knowledge of the local teams outside of the hospital setting enabled these links to work effectively.

#### Model 4-palliative care

In response to care needs, this model was in place for those needing palliative care. Even for key workers who were mainly hospital based, this might change where there was a greater need for involvement at home during palliative and end of life care, and in some cases these visits were daily:

Certainly for the patients needing palliative care we’re very much in the driving seat in terms of assessment of patients’ needs both from a medical, psychological and emotional perspective, looking at drug interventions and the whole co-ordination of their care linking in with general practitioners and local services, children’s hospices (Key Worker 9, interview)

Evaluation/performance data showed the increase in meetings, and phone contacts with these families, again this was often daily and some key workers also offered a 24 hour on call service for patients who were palliative. The key worker discussed with parents the options for end of life care, what they could expect and would support parents in their choice, for example, contacting the hospice, organizing a visit before the parents decided; meeting with the different professionals involved in this phase, this was much valued by parents:

(key worker) held it all together, the hospital team, the medical team, the community … she connected with everyone, the social workers, the play therapist, she was like the important centre of the team (Bereaved Parent 8, interview)

Across these models, all key workers were involved in care coordination, but not all were involved in direct care delivery; ward nurses or children’s community nurses were more likely to be called upon for this. Balancing care coordination and direct clinical care was explored more in the focus groups. Revealing a shared understanding that other health professionals might be involved in the delivery of treatments, but key workers maintained their role with families, providing information, practical and emotional support and specialist advice:

I think that’s really important, that we don’t just provide clinical care as being, going and putting up some chemo or taking-, good clinical care (…) it’s subtle, using all of your expertise, all of your knowledge and skills in every single interaction that you have with the family. So, you may not be going out putting up chemo but you’re certainly still doing clinical care. (Key Worker, focus group)

Models of care adopted was influenced by resources available, both in the community and in the principal treatment centres. The context of care influenced the key worker role, and revealed variation in roles and responsibilities.

### Context of care

The emphasis of the role was upon patient and family holistic assessment, supporting families, education, providing information and continuity of care within a multidisciplinary team (MDT) framework. The role in practice varied, described earlier within the four models. These models were clearly reflective of service need, the context facilitated the delivery of the role, but it also made it more challenging. This was linked to a number of contextual factors, and these varied between services.

#### Contextual facilitators

These included the following: good collaboration between services; good communication, and keeping all professionals in the different services informed; being clear about each other’s role; well-established knowledge about the principal treatment centres and availability of local teams’ resources and expertise. Coordination of care was facilitated by key workers knowing the local teams and what was available in the community. For example, sometimes services were not very local to families or there was limited capacity and reduction in the services available or even a lack of availability of services; key worker knowledge of this local context was always evident.

#### Contextual barriers

As identified by professionals, roles were hindered where there was separate documentation in use, a lack of awareness and appreciation of the roles and responsibilities of others between services and potential overlap of the roles and duplication of services. The context was also challenging due to (pressure on) time, and the individual nature of key workers caseload size, geographical area, staffing numbers and the inequality of local services’ resources. Increased caseloads (resulting from staff maternity leave, retirement; lack of cover) and/or large caseload led to pressures on the key worker and service delivery (although the median number of patients per caseload was between 39 to 51 cases, some key workers had double this number):

It is the same, because there are 150 families I think, and just me still. So nothing has changed. So there will be days where it is quiet, and they will be able to get through on the first ring, and there will be other days where, almost by the time they get through, you know, they are, ‘Oh, at last, we’ve got through.’ They always know if their children are unwell that they have to ring the local hospital, and actually, possibly trying to get hold of me would get in the way of that. (Key worker 16, interview)

Thus practical/clinical aspects of the role had to be prioritized, leaving less time to provide emotional support for families. In these cases, the role became more administrative-focused, with little time for face-to-face contact with families. Services were perceived as inequitable, with a focus on newly diagnosed patients and/or those with complex needs. This had an impact on families’ experience:

(…) there isn’t that same level of support when you move into the chronic phase, I think. That would be the only gap, I would say (Parent 3, interview)

once you get to the end of treatment, I personally felt quite dumped by the system (Parent 20, interview)

### Process of care

Key workers expressed a shared view of their role. The role was characterized by a number of consistent defining attributes, that helped them to perform their role, these included, being organized; having good time management; willing to seek advice from others; being able to signpost; having an expert understanding of the treatment process; being very experienced nurses; knowledgeable of the organization; having an expanded understanding of how care is delivered in the community. In addition, attributes such as, being friendly, and a good communicator; being approachable, nice, empathetic, supportive, and compassionate; having a good sense of humour; being an advocate; and “able to say no”, as reflected here by one child:

I thought it was a brilliant thing to have because it just kept some easy to access links to what we were going through because [the key worker] knew what we needed to do in certain situations. [The key worker] was always there to sort out any worries or questions we had for her. [The key worker] was just really useful and really nice. (Child 4, interview)

Underpinning the role, was the importance of working in partnership with families. It was felt that this decreased parents’ feelings of isolation and increased their confidence.

Key working effectiveness in their role was built upon three pillars, care coordination, expert knowledge, expertise and experience, and relationships. In the data these pillars were interlinked, but are described here as discrete elements to highlight more clearly the process of care.

### *Care coordination -“*well, I’d call it knitting”

Key workers were the main connecting role in the principal treatment centres; there was a clear pathway, enabling key workers to streamline the communication process between families and professionals and between professionals:

a first point of contact, and, actually, because we’ve all got nursing backgrounds, then we understand a) the treatment process, and we understand the organization (…) being able to talk to somebody who understood the complexities of a hospital, basically, and, actually, there are so many members of the team. You don’t want to have to keep on going round the houses to try and get to the people you want to, kind of, speak to (Key worker 3, interview)

The majority of professionals agreed that the key worker improved coordination of care (86%, n = 73). They simplified the contacts between services (80%, n = 68), improved information sharing with and between professionals (78%, n = 66) and families (82%, n = 70), improving the families overall experience of care (80%, n = 68; Table IV).

**Table IV.** Professionals’ views on the key worker role (from questionnaires).

The key worker role	Disagree n (%)	Neutral n (%)	Agree n (%)	Don’t Know n (%)	Missing data n (%)
Improved coordination of care	3 (4%)	4 (5%)	73 (86%)	4 (5%)	1 (1%)
Simplified the contacts between services	3 (4%)	7 (8%)	68 (80%)	5 (6%)	2 (2%)
Improved information sharing between professionals	6 (7%)	10 (12%)	66 (78%)	2 (2%)	1 (1%)
Improved information sharing with families	4 (5%)	7 (8%)	70 (82%)	3 (4%)	1 (1%)
Enable care closer to home	9 (11%)	12 (14%)	50 (59%)	13 (15%)	1 (1%)
Improved families experiences	3 (4%)	4 (5%)	68 (80%)	9 (11%)	1 (1%)

To be the main point of contact, key workers needed to know the families well—families’ needs assessment was central to this. Families’ needs are known to be wide ranging and likely to change during treatment; hence key workers worked holistically and logically when assessing and reassessing needs. In the majority of cases, this was informal, as opposed to being based on the use of any validated instrument, more based on their holistic needs assessment. Key workers had frequent contact with education, social services, other relevant local organizations and voluntary agencies, and this was informed by a holistic approach to family needs that was grounded in an MDT approach:

You’re kind of this person in the middle who’s with the family, who tries to coordinate a whole lot of other stuff that kind of goes along with the diagnosis of cancer (…) Lots of professionals get involved, and for families that can be very confusing, and they don’t know quite who is doing what where. To have one person that they feel they can contact (Key Worker 8, interview)

The key worker coordinated care closer to home, reducing the time in hospital and the disruption of the child’s life. They had an important role in the transition back to school, working with the child, family and school to plan and support this. They did this by, for example, going to school meetings; ensuring all equipment and support was in place; ensuring that staff felt confident and knew how to support the child in their care.

Parents indicated that, in the majority, the key worker fulfilled core aspects of the role, including: coordination of care, speaking on behalf of the family, giving advice, providing information about the child’s condition and helping in a crisis. There was less agreement in terms of them being able to “Identify the needs of all the family members”; 35% (n = 33) said “some” and 27% (n = 26) said “not at all” and “address the needs of all family members”; 32% (n = 30) said “some” and 32% (n = 30) said “not at all” (Table V).

**Table V.** How much the key worker fulfilled aspects of the role (from questionnaires).

	Not at all n (%)	Some n (%)	Very much n (%)	Missing data n (%)
Emotional support	16 (17%)	35 (37%)	41 (43%)	3 (3%)
Information about your child’s condition	13 (14%)	28 (30%)	51 (54%)	3 (3%)
Information about services	16 (17%)	32 (34%)	43 (45%)	4 (4%)
Advice	8 (8%)	29 (31%)	55 (58%)	3 (3%)
Identifying the needs of all family members	26 (27%)	33 (35%)	30 (32%)	6 (6%)
Addressing the needs of all family members	30 (32%)	30 (32%)	29 (31%)	6 (6%)
Speaking on behalf of the family when dealing with services	24 (25%)	19 (20%)	47 (50%)	5 (5%)
Coordinating care	13 (14%)	29 (31%)	47 (50%)	6 (6%)
Improving access to services	22 (23%)	22 (23%)	41 (43%)	10 (11%)
Help/support in a crisis	21 (22%)	20 (21%)	48 (51%)	6 (6%)
Signposting you to services	21 (22%)	29 (31%)	39 (41%)	6 (6%)

Professionals acknowledged that the key worker was often responsible for making contact with members of the community MDT (54%, n = 46 always; 24%, n = 20 frequently), ensuring the sharing of appropriate information across agencies and key people involved in the delivery of care (48%, n = 41 always; 32%, n = 27 frequently) as well as being the single point of contact for relevant services involved (38%, n = 32 always; 38%, n = 32 frequently; Table VI).

**Table VI.** Professionals’ perspective of the key worker coordinating role (from questionnaires).

	Never n (%)	Rarely n (%)	Sometimes n (%)	Frequently n (%)	Always n (%)	Don’t know n (%)	Missing data n (%)
Being the single point of contact and named individual for the relevant services involved	0 (0%)	4 (5%)	10 (12%)	32 (38%)	32 (38%)	5 (6%)	2 (2%)
Coordination of input from other members of the MDT into the assessment and care planning process	1 (1%)	6 (7%)	10 (12%)	25 (29%)	38 (45%)	3 (4%)	2 (2%)
Making contact with community practitioners/members of the community MDT upon the child’s discharge from the principal treatment centre and when significant care needs change	0 (0%)	5 (6%)	6 (7%)	20 (24%)	46 (54%)	6 (7%)	2 (2%)
Ensuring the sharing of appropriate information across agencies and key people involved in delivering care	1 (1%)	4 (5%)	9 (11%)	27 (32%)	41 (48%)	1 (1%)	2 (2%)
Responsible for coordinating a community MDT when required by engaging with professionals in primary care and establishing robust channels of communication	2 (2%)	7 (8%)	8 (9%)	21 (25%)	36 (42%)	9 (11%)	2 (2%)

The majority of parents reported a positive experience; there were however parents whose experiences were less positive. Results show that 59% (n = 56) of parents were very satisfied with the key worker service they received, 19% (n = 18) were satisfied, 11% (n = 10) were not satisfied and 5% (n = 5) were not at all satisfied with the service. For example, one parent shared in their interview that the lack of communication and information meant they had to “chase around” for information and coordinate support without any help. They also reported feeling at times they did not know what to expect due to lack of information sharing and insufficient support with the child’s transition back to school. Another parent reported a similar stressful experience, but this changed when a key worker role was in place, the experience improved when they had a key worker coordinating care, informing, and supporting them. There was also a significant improvement of the child’s experience when the key worker started working with the family (e.g., increased adherence with procedures; reduced stress levels):

We wouldn’t have managed without them (…) you’re led through this maze and without somebody to guide you through it, you’d be stumbling all the way (Parent 12, interview)

Coordination of care, needs assessment and care planning facilitated care closer to home, saving the organization and families’ resources (e.g., reducing preventable admissions).

### *Knowledge, expertise and experience—*“they could just explain other details that you’d not fully understood to you”

Expertise and experience was core to the role and was valued by all the professionals in our study. One of the main advantages of the role was their knowledge and skills, this was shared with other professionals formally on study days, training sessions in the hospital and in the community and informally in MDT meetings. It was emphasized how key workers were an approachable and expert source of support for families; facilitating care closer to home and reducing visits to the hospital. The role was perceived as an excellent asset to maintain and improve quality of care.

Key workers supported parents understanding information about diagnosis, treatment plans, protocols and the varied professionals involved in the child’s care. One parent described how the key worker was present when they were informed their child had relapsed and could support them afterwards with understanding the information shared:

which was really important, actually, because emotionally we were all over the place. (Key worker) was somebody that we knew we could trust to hear the information that we needed to hear and to be able to then have a conversation with them outside that, kind of, tense time, for (key worker) to be able to just go through the options with us of what was available (Parent 3, interview)

Around half the parents reported getting enough advice about “learning the best ways of helping my child” (48%, n = 46) and “having someone to talk about my child with” (50%, n = 47). Advice was needed to: “help with planning for my child’s future” (36%, n = 34) and “having someone who will show us which services are available to us” (32%, n = 30; Table VII).

**Table VII.** Parental needs (from questionnaires).

	Getting enough advice n (%)	Need advice n (%)	Advice not needed n (%)	Missing data n (%)
Help with managing my child’s behaviour	26 (27%)	16 (17%)	48 (51%)	5 (5%)
Help getting my child to sleep better	17 (18%)	12 (13%)	62 (65%)	4 (4%)
Learning the best ways of helping my child	46 (48%)	22 (23%)	23 (24%)	4 (4%)
Having someone to talk about my child with	47 (50%)	19 (20%)	25 (26%)	4 (4%)
Help with the day to day care of my child	27 (28%)	9 (10%)	53 (56%)	6 (6%)
Having someone who will show us which services are available to us	40 (42%)	30 (32%)	20 (21%)	5 (5%)
Meeting other parents of children with cancer	27 (28%)	17 (18%)	46 (48%)	5 (5%)
Help with planning for my child’s future	28 (30%)	34 (36%)	26 (27%)	7 (7%)
Help getting the information we need	37 (39%)	22 (23%)	32 (34%)	4 (4%)
Help planning my child’s schooling	39 (41%)	23 (24%)	29 (31%)	4 (4%)
More time to spend with my child (e.g., to play)	14 (15%)	9 (10%)	67 (71%)	5 (5%)
Help obtaining aids and equipment for my child	28 (30%)	9 (10%)	50 (53%)	8 (8%)
Getting a break from caring for my child	9 (10%)	11 (12%)	70 (74%)	5 (5%)
Spending more time with my partner	5 (5%)	13 (14%)	72 (76%)	5 (5%)
Having more time with my other children	8 (8%)	16 (17%)	63 (66%)	8 (8%)
Help with the housework	4 (4%)	9 (10%)	77 (81%)	5 (5%)
Having more money in order to care for my child	29 (31%)	18 (19%)	43 (45%)	5 (5%)
Help with my child during the school holidays	6 (6%)	12 (13%)	73 (77%)	4 (4%)
Having someone to look after my child so I can go to work	5 (5%)	11 (12%)	74 (78%)	5 (5%)
Help with transport problems	9 (10%)	7 (7%)	75 (79%)	4 (4%)

Key workers also played an important role in the child’s understanding of their condition and information shared. Understanding this information was important to children as it made them feel less worried and scared:

[the key worker] was there to just discuss things with that you wouldn’t normally get the information off the doctor straight. They could explain the information that the doctor gave to you or they could just explain other details that you’d not fully understood to you (Child 4, interview)

Children also shared how the lack of the key worker support could impact their experience, for example, it could mean not having a professional with the time to discuss their concerns.

### *Relationship -*“you have one person who knows you”

The relationship was built gradually over time and through the different stages of their journey. Support and contact with families were described as intense at diagnosis: a phase key workers and families both highlighted as crucial. At diagnosis, families have to deal with complex information and the emotional impact of their child’s diagnosis. Key workers discussed information about the diagnosis, treatment and helped the family understand the information shared (e.g., explaining the medical terminology and plan for treatment). Families had many questions and appreciated the opportunity to be able to ask someone with expertise who took time to listen to them. Parents’ reported the key worker had reduced their stress levels (56%, n = 53) and had an impact on their peace of mind (being less worried [56%, n = 53]) and emotional/mental health (39%, n = 37).

Variation in experience was more apparent following the diagnosis phase. Entering the treatment phase, experiences and needs differed depending upon the child’s diagnosis and treatment protocol. In common, however, was the need for continuity and consistency of care, particularly at transition points: from hospital to home; from principal treatment centre to local hospital; between teams/professionals. Consistency and continuity were facilitated by the relationship established between the key worker and family. In addition to the face-to-face contact in the hospital or at home, families contacted the key worker via phone calls, text messages and email. The provision of contact details and how this message was communicated legitimized contact. Key workers were seen as approachable and parents felt confident in contacting them. Parents sought advice when uncertain and key workers, with their expertise and knowledge, could advise parents, monitor any changes and develop a plan of action; as a result being able to contact the key worker facilitated children staying at home safely.

Continuity of care was also facilitated by the way the key worker managed and informed the parent about the transition back home and the support available (e.g., if community teams were involved, key workers facilitated parents’ confidence in the new professionals). Notwithstanding parents’ desire to go home, some feared caring for their child at home, so being able to go home with support was highly valued by parents. Home visits were seen as a mechanism that facilitated transition back home and the relationship with families, as the key worker had the opportunity to see the family in their environment. Parents also reported feeling more comfortable to talk about their concerns at home:

I think the most important time is when you start that process of leaving hospital (…) it’s quite a big transition (…) While you have somebody in the ward, just looking after your every need and then you’re at home and you’re having to do it all, so that was a really important time that [key worker name] would come (…) and sit and listen, that would, I say, was highly important, in terms of just being at home and trying to transition from hospital to home (…) (Parent 3, interview)

Although key workers supported parents’ empowerment, parents’ reported that for 21%, (n = 20) the key worker did not act as an advocate and help them develop their self-advocacy skills; for 16% (n = 15) the key worker did not help them to know about how to access the services and 14% (n = 13) were not supported to increasingly take on coordination of their child’s care (Table VIII).

**Table VIII.** Parents empowerment (from questionnaires).

	Never n (%)	Sometimes n (%)	Often n (%)	Always n (%)	Not applicable n (%)	Missing data n (%)
Helped you know about and how to access the services	15 (16%)	22 (23%)	19 (20%)	22 (23%)	12 (13%)	5 (5%)
Acted as an advocate and helped you to develop your self-advocacy skills	20 (21%)	16 (17%)	12 (13%)	21 (22%)	20 (21%)	6 (6%)
Supported and enabled you where possible to increasingly take on coordination of your child’s care	13 (14%)	18 (19%)	19 (20%)	20 (21%)	19 (20%)	6 (6%)

On the whole, parents, children and key workers’ descriptions of the support given and received showed that the impact on families’ experiences was grounded in the relationship established with the family, as illustrated here:

It’s like a relationship, a trust, and that’s what we’ve got with the key worker (Bereaved Parent 2, interview)

[key worker] knew how to help, like, how to, the right stuff to do for me (…) [key worker] knew how to help my Mum as well (…) Coping. I mean, like, we’re coping with the fact that I actually have cancer (…) [key worker] talked to her a lot (Child 3, interview)

## Discussion

In this study, we sought to understand the complexities of the key worker role in children’s cancer care. We wanted to fully understand the elements of the role that could then support organizations to improve/refine existing roles, or to develop future key worker models of care, for populations other than those with cancer. We identified that coordination, consistency and continuity of care facilitated meaningful outcomes for families. We demonstrated that these processes of care were enabled in a context where the key worker had significant experience and expertise, had the appropriate resources, with a manageable caseload, across a realistic geographical area that together facilitated good relationships within and outside of the principal treatment centres. In this context, families reported positive outcomes, and a helpful relationship from diagnosis, and beyond completion of therapy. Where the context shifted, where resources were limited, and the key worker had a large caseload, across a significant geographical area, that then limited opportunity to develop meaningful professional relationships, particularly outside of the principal treatment centres. In this context, families reported a more tangible focus on the early part of their cancer trajectory, with transition points, such as end of treatment, feeling less supported. We examine our findings, drawing upon published work, to highlight the essential features of coordination, consistency and continuity of care that influence the successful implementation of the key worker role.

When care is well coordinated patients will experience effective flow of information between clinicians throughout the course of their illness, with streamlined service provision in response to their physical, emotional and social needs. The capacity of key workers to perform coordination of care required the key worker role to be adequately supported. We found that the successful implementation and sustainability of the role was influenced by a shared understanding within the teams working in the various hospitals and community about the key worker role and the knowledge and expertise of the key worker about the services provided by other teams. The outcomes from fragmented care and the benefits of care coordination are well described (Simpson et al., 2021). Reinforced in our study, having a named contact person was a key component of care coordination (Freijser et al., 2015). To be successful, this needed to encompass, “frequent, timely, problem-solving communication, supported by relationships of shared goals, shared knowledge and mutual respect” (Gittell, 2006, p. 85). These characteristics were at the core of the key worker role when it worked best, enriching patient and family experience.

Time constraints are already known to hinder the implementation of the key worker role (Greco et al., 2005). Similar to Sloper et al.’s (2006) study, coordination of care and family support were only possible if key workers had time to fulfil all aspects of their role. In addition to time, key workers needed resources for administrative support. This had an impact on the time available for contact with families and services and it was seen by key workers as an additional task that was not the best use of their skills; reported previously in adult cancer care (Ling et al., 2017). The success of the role was dependent on the support structures around it. Our study showed parents and children valued having a consistent point of contact, someone who knew them and had the expertise to support them. This consistency and continuity of care, valued so much by families, had consequences. Managing parent and family expectations, and the need to maintain consistency and continuity of care at defined transition points, for example, from hospital to home, from principal treatment centres to local hospital and between teams/professionals, added to the emotional burden of care. The impact of this, and the emotionally charged interactions with families has been reported previously (Hillis et al., 2016). However, it is this relationship with families and professionals that is so central to this role. Maintaining professional boundaries therefore becomes critical. Important factors for implementing successful key working included managing the relationship between the key worker the parents and their children.

Parents placed value on a professional that knew them well, knew what they have been through, and knew the family and their child best. This relational continuity, characterized by an on-going therapeutic relationship between a patient and one or more providers, is just one type of continuity families prized. Management and informational continuity, described by (Haggerty et al., 2003), were also highly valued. Key worker’s expertise and experience were at the core of maintaining all types of continuity. Similar to others, our work shows that this continuity, that involved emotional, educational and practical support, was at its best when experienced, clinical nurse specialists were performing this role (Kerr et al., 2021). Coherent, connected and consistent care was delivered by these experts. They enabled the development of local teams’ competencies and consequently made possible care closer to home (Parker et al., 2012). This contributed to parents’ education and confidence to feel safe at home with their child. Being able to go home, regain a sense of normality and being able to be with other family members and to have contact with friends, were all made possible through this model of care. A further aspect of care closer to home enabled children to participate in their own education. Similar to children’s community nursing models of care, key workers in cancer care, worked closely with schools to plan and support the safe reintegration of children into school (NHS Benchmarking Network, 2020; Robson & Beattie, 2004). Supporting children who have continuing health needs to continue with their education is essential, to promote better education outcomes (Burns et al., 2021). Preserving normality in a child’s daily life, and maintaining their social development, significant in parenting an ill child (McEvoy & Creaner, 2021), were central to the key worker role.

### Relevance to clinical practice

Our findings support the notion that key working effectiveness was dependent upon three pillars: coordination; knowledge, expertise and experience; and relationship. But in certain conditions, such as a variation in context, this placed limits on enacting the three pillars, and the positive experience of the family was diminished. When they worked well, key worker roles reduced the fragmented nature of services, families placed a great value on their role, wanting to retain the same key worker from diagnosis, beyond treatment into long-term care. Retaining these roles, where they are already in place, or including, if not, we would highly recommend, factoring into budgets to sustain such roles. Service need influenced the type of key worker model in place, our findings enrich these models, and evidence the importance of clinical teams describing what is needed for their patients, in terms of coordination, consistency and continuity in care. Variations in model delivery should be welcomed, but need to be evaluated, to ensure patient outcomes, patient experience, and equity of access to services remains.

### Limitations

The study has limitations that should be considered when interpreting the findings. The data represents a snapshot in time of views of the key worker role and this role is continuing to develop and change. Future evaluations would be welcomed, in the light of any reconfiguration of cancer services, we would recommend maintaining a focus on family experience, with the child at the centre, but with the addition of economic evaluation, to further aid replicating successful services (Jessup et al., 2020). Similar to other work, information about caseloads, case mix and costs require further description (Parker et al., 2012). In addition, this is a reflection on the key worker role within children’s cancer care in the UK, as such the findings cannot directly be generalized to other contexts or populations. However, as the findings concur with previous work focusing on different patient populations in different countries (Chollette et al., 2020), we might suggest that such resonance indicates similarities which would be evident in other services employing key worker/care coordinator type roles.

## Conclusion

The key worker role has been firmly established in principal treatment centres across England, Wales and Scotland. We have described here the core, and in the main shared characteristics, as well as the differences, more associated with different models of implementation and service requirements. Similar to others, we have shown the role to be complex and diverse, responding to local needs. Knowledge, experience and expertise, coordination and relationship, the three pillars, were essential to an improved family experience, emotional wellbeing and the delivery of care closer to home. When these pillars where in place, the key worker role could make a positive contribution to a better quality family experience, with care individualized and parents reported not feeling as “just another case”.

## Supplementary Material

Supplemental Material
