# Supplementary material for: Care coordination, consistency and continuity: the case of the key worker role in children’s cancer care
Source: Int J Qual Stud Health Well-being. 2022 Jun 26;17(1):2092958. doi: 10.1080/17482631.2022.2092958 (PMC9246033; doi:10.1080/17482631.2022.2092958)
Supplement: Supplemental Material [file ZQHW_A_2092958_SM9714.docx]

**Supplementary file 1**

Good Reporting of A Mixed Methods Study (O'Cathain, Murphy, & Nicholl, 2008)

| 1 | Describe the justification for using a mixed methods approach to the research question | ✓ | In the methods section (from page 5) |
| --- | --- | --- | --- |
| 2 | Describe the design in terms of the purpose, priority and sequence of methods | ✓ | In the methods section (from page 5) |
| 3 | Describe each method in terms of sampling, data collection and analysis | ✓ | In the methods section (from page 5) |
| 4 | Describe where integration has occurred, how it has occurred and who has participated in it | ✓ | In the methods (from page 5)/results (from page 8)/discussion  (from page 20) sections |
| 5 | Describe any limitation of one method associated with the present of the other method | ✓ | Both methods enhanced the study |
| 6 | Describe any insights gained from mixing or integrating methods | ✓ | In the discussion  section (from page 20) |

O'Cathain, A., Murphy, E., & Nicholl, J. (2008). The quality of mixed methods studies in health services research. *Journal of Health Services Research and Policy, 13*(2), 92-98. doi:10.1258/jhsrp.2007.007074
